# The number of prehospital defibrillation shocks and 1-month survival in patients with out-of-hospital cardiac arrest

**DOI:** 10.1186/s13049-015-0112-4

**Published:** 2015-04-17

**Authors:** Manabu Hasegawa, Takeru Abe, Takashi Nagata, Daisuke Onozuka, Akihito Hagihara

**Affiliations:** Guidance of Medical Service Division, Health Policy Bureau, Ministry of Health, Labour and Welfare, 2-2 Kasumigaseki 1-chome, Chiyoda-ku, Tokyo 100-8916 Japan; Medical Center, Yokohama City University, 4-57 Urafunecho, Minami-ku, Yokohama, Kanagawa 232-0024 Japan; Kyushu University Hospital, Department of Emergency and Critical Care Center, Higashi-ku, Fukuoka, 812-8582 Japan; Kyushu University Graduate School of Medicine, Department of Health Services Management and Policy, Higashi-ku, Fukuoka, 812-8582 Japan

## Abstract

**Background:**

The relationship between the number of pre-hospital defibrillation shocks and treatment outcome in patients with out-of-hospital cardiac arrest (OHCA) presenting with ventricular fibrillation (VF) is unknown currently. We examined the association between the number of pre-hospitalization defibrillation shocks and 1-month survival in OHCA patients.

**Methods:**

We conducted a prospective observational study using national registry data obtained from patients with OHCA between January 1, 2009 and December 31, 2012 in Japan. The study subjects were ≥ 18–110 years of age, had suffered from an OHCA before arrival of EMS personnel, had a witnessed collapse, had an initial rhythm that was shockable [VF/ventricular tachycardia (pulseless VT)], were not delivered a shock using a public automated external defibrillator (AED), received one or more shocks using a biphasic defibrillator by EMS personnel, and were transported to a medical institution between January 1, 2009 and December 31, 2012. There were 20,851 OHCA cases which met the inclusion criteria during the study period. Signal detection analysis was used to identify the cutoff point in the number of prehospital defibrillation shocks most closely related to one-month survival. Variables related to the number of defibrillations or one-month survival in OHCA were identified using multiple logistic regression analysis.

**Results:**

A cutoff point in the number of pre-hospital defibrillation shocks most closely associated with 1-month OHCA survival was between two and three (*χ*^*2*^ = 209.61, *p* < 0.0001). Among those patients who received two shocks or less, 34.48% survived for at least 1 month, compared with 24.75% of those who received three shocks or more. The number of defibrillations (odds ratio [OR] = 1.19, 95% CI: 1.03, 1.38), OHCA origin (OR = 2.81, 95% CI: 2.26, 3.49), use of ALS devices (OR = 0.68, 95% CI: 0.59, 0.79), use of epinephrine (OR = 0.33, 95% C: 0.28, 0.39), interval between first defibrillation and first ROSC (OR = 1.45, 95% CI: 1.18, 1.78), and chest compression (OR = 1.21, 95% CI: 1.06, 1.38) were associated significantly with 1-month OCHA survival.

**Conclusions:**

The cutoff point in the number of defibrillations of patients with OHCA most closely related to one-month survival was between 2 and 3, and the likelihood of non-survival 1 month after an OHCA was increased when ≥3 shocks were needed. Further studies are needed to verify this finding.

**Electronic supplementary material:**

The online version of this article (doi:10.1186/s13049-015-0112-4) contains supplementary material, which is available to authorized users.

## Background

Defibrillation is an important intervention for patients with out-of-hospital cardiac arrest (OHCA) during advanced life support (ALS). In particular, treatment combining chest compressions with defibrillation is recommended when a patient presents with ventricular fibrillation (VF). As recurrence of VF after the first fibrillation is common, two or more defibrillation shocks are necessary typically [1-3]. Regarding the manner in which defibrillation and chest compressions should be coordinated during pre-hospital cardiopulmonary resuscitation (CPR) in an OHCA patients, the CPR guidelines released in 2005 recommended resuming CPR immediately for 2 consecutive min following a defibrillation shock to minimize the CPR “hands-off” time [4,5]. In addition, each shock should be followed by a rhythm analysis and pulse check after the 2-min CPR. The current (2010) and 2005 CPR guidelines are in accordance with regard to pre-hospital defibrillation [6].

Data pertaining to the response of recurrent shockable rhythm episodes to defibrillation shock are limited [1,2,7]. According to a Dutch study using the resuscitation guidelines of 2000, the rate of VF termination by defibrillation was 92% following the first shock, compared with 61% after the second shock and 83% after the third shock [7]. However, 48% of the cohort presented refibrillation within 2 min of the first defibrillation shock, and 74% received at least one shock for refibrillation during the prehospital ALS process [7]. According to the current guidelines, when two or more shocks are required, a shock should be administered following CPR (for 2 consecutive min), a rhythm analysis, and pulse check. Furthermore, and particularly in Japan, the second, and any subsequent, shocks should be administered under the guidance of an on-line physician [8,9]. Therefore, it takes longer to deliver a shock in Japan than in areas where permission from an on-line emergency physician is not required.

If three shocks were allowed without the approval of an on-line physician, an improved resuscitation outcome in the OHCA would be expected. At present, the relationship between the number of defibrillation shocks given to OHCA patients presenting with a shockable rhythm in pre-hospital and 1-month survival is unknown. The purpose of this study was to assess the association between the number of pre-hospital defibrillation shocks and 1-month survival in OHCA patients. This was a prospective observational study using national Japanese registry data.

## Methods

### Data collection

The emergency medical service (EMS) system in Japan has been explained previously [10,11]. Briefly, in 2011 Japan had a population of 127,959,771, and EMS was provided through 798 fire stations with municipal government dispatch centers [12]. Except for obvious death, such as decapitation, incineration, decomposition, rigor mortis, or dependent cyanosis, all patients with OHCA who are treated by EMS personnel are transported to hospitals, because Japanese guidelines prohibit terminating resuscitation in the field [8]. In most cases, an ambulance crew consisted of three emergency personnel, including at least one emergency life-saving technician. The current Japanese CPR guidelines, which are based completely on the AHA 2005 CPR guidelines, allow only one defibrillation shock; an EMS crew must obtain approval from an on-line emergency physician when two or more shocks are required to treat VF [9]. Using the standardized Utstein style template, all OHCA cases were registered in a prospective, nationwide, population-based database by the Fire and Disaster Management Agency (FDMA). The EMS person in charge of each OHCA patient contacted the doctor who treated the patient to collect 1-month follow-up data [8]. After an electronic data check by the FDMA, data from the 798 fire stations in the 47 prefectures were integrated into a national registry system on the FDMA database server.

This study was reviewed and approved by the ethics committee at Kyushu University Graduate School of Medicine. The requirement for written informed consent was waived.

### Subjects

**Figure 1:**
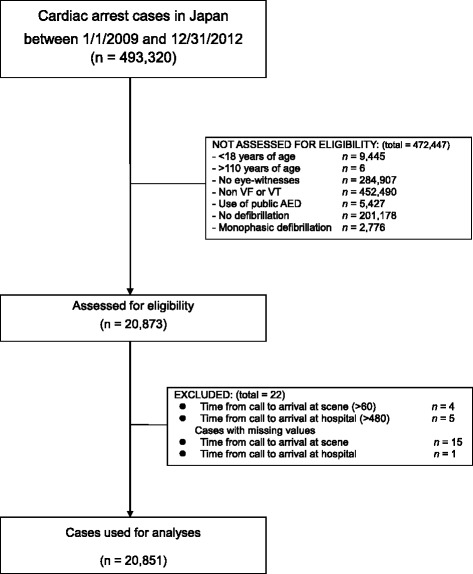
The out-of-hospital cardiac arrest (OHCA) cases evaluated for the analysis. The number of each item in the “NOT ASSESSED FOR ELIGIBILITY” and “EXCLUDED” boxes does not sum up to the total numbers in the boxes due to overlapping items.

Study subjects in 2009 were resuscitated under the 2005 resuscitation guidelines, and those in 2010, 2011 and 2012 were resuscitated under the 2010 resuscitation guidelines. The study patients were ≥ 18–110 years of age, had suffered from an OHCA before arrival of EMS personnel, had a witnessed collapse, had an initial rhythm that was shockable [VF/ventricular tachycardia (pulseless VT)], were not delivered a shock using a public automated external defibrillator (AED), received one or more shocks using a biphasic defibrillator by EMS personnel, and were transported to a medical institution between January 1, 2009 and December 31, 2012. Because this study aimed to evaluate the association between the number of pre-hospital defibrillations (administered by EMS personnel using a biphasic defibrillator) and 1-month survival after an OHCA, patients administered a shock with a public AED were excluded from the analysis. In addition, patients for whom the time from the call until arrival at the scene was > 60 min or from the call until arrival at the hospital was > 480 min were excluded from the analysis. Of 493,320 OHCA cases between January 1, 2009 and December 31, 2012, 20,851 (4.23%) were used for analysis, and the remaining cases were excluded according to the inclusion criteria (Figure 1). It is notable that >95% of the initial subjects (n = 493,320) were excluded due to multiple inclusion criteria. Since information on a patient’s status 1 month after the event is mandatory in the Utstein template, no patients were lost to follow-up in the study.

### Study variables

The study variables are listed in Table 1. The origin of cardiac arrest (i.e., cardiac or noncardiac) was determined clinically by the physician in charge with the aid of EMS personnel. The shockable rhythm category also included pulseless VT. Time from the call to the scene or until arrival at the hospital was measured using fire station dispatch records and the watch of an emergency lifesaving technician. The endpoint was 1-month survival after cardiac arrest. In addition, to describe the neurologic status of survivors, survival with minimal neurological impairment, which was defined as a cerebral performance category (CPC) category of 1 or 2, and survival with minimal neurological impairment, which was defined as an overall performance category (OPC) of 1 or 2, were calculated [13,14]. The mean CPC or OPC among survivors was also calculated. The EMS person in charge of each OHCA patient had a face-to-face consultation with the doctor who treated the patient at the hospital to collect the 1-month follow-up data. If the patient was no longer at the hospital, the EMS personnel conducted a follow-up search, and 1-month survival data was collected after the event.

**Table 1:** **Baseline characteristics of the patients with out-of-hospital cardiac arrest (OHCA) who received a biphasic defibrillation shock before hospital arrival (n = 20,851)**

**Variables**	
(OHCA patients)	
Age (yr) (mean ± standard deviation [SD])	65.73 ± 15.28
Sex (male), no. (%)	16368 (78.50)
Origin of OHCA (cardiac origin), no. (%)	18598 (89.19)
Relationship between bystander and patient (family member), no. (%)	11194 (53.69)
(CPR initiated by bystander)	
Chest compressions (yes), no. (%)	9681 (46.43)
Rescue breathing (yes), no. (%)	2228 (10.69)
(Life support by EMS personnel)	
Emergency life-saving technician in ambulance (yes), no. (%)	20284 (97.28)
Advanced life support by MD (yes), no. (%)	2648 (12.70)
Time from call to arrival at scene (min) (mean ± SD)	7.09 ± 3.25
Time from call to arrival at hospital (min) (mean ± SD)	33.68 ± 15.50
Use of ALS devices (laryngeal mask/an adjunct airway/ tracheal tubes), no. (%)	8440 (40.48)
Epinephrine use (yes), no. (%)	4644 (22.27)
Time from the first defibrillation to the first ROSC before hospital arrival (min) (mean ± SD)	7.67 ± 9.59
Number of defibrillations by EMS personnel (mean ± SD)	2.36 ± 1.67
Proportions of the number of defibrillations (%)	
1	8403 (40.30)
≥2	12448 (59.70)
Frequency of patients who took ≤1 min. from the first defibrillation to the first ROSC before hospital arrival (%)	2743 (13.16)
(Endpoint)	
1-month survival after cardiac arrest (yes), no. (%)	6480 (31.08)
ROSC (yes), no. (%)	6876 (32.98)

### Statistical analysis

The data from patients who experienced OHCAs between January 1, 2009 and December 31, 2012 in Japan and were in the national registry (n = 493,320) and who met the inclusion criteria regarding patient age, treatment, and time course were analyzed (n = 20,851) (Figure 1). Descriptive analyses using the entire sample set were conducted using t-tests for continuous variables or chi-square tests for categorical variables.

Signal detection analysis (SDA) (ROC 5.0 software19) was performed to determine the cutoff point in the number of prehospital defibrillation shocks which is most closely related to one-month survival in ROSC. SDA focuses on the parameters of “sensitivity” and “specificity” [15,16]. “Sensitivity” was defined as “the percentage of patients who had a specific number of defibrillation shocks among those who survived 1 month”. “Specificity” was defined as “the percentage of patients who did not have a specific number of defibrillation shocks among those who did not survive 1 month”. This signal detection parameter is equivalent to the χ2 statistic (df = 1), which means that the subjects are categorized into a 2 × 2 table consisting of dependent and independent variables [15,16]. SDA is called repetitive partitioning, and “number of defibrillation shocks” had nine cut-off points (i.e., 1, 2, 3, 4, 5, 6, 7, 8, 9, 10, or more). When the parameter (i.e., number of shocks) has the largest χ^2^ value (df = 1) at a certain cut-off point, the cutoff point in the equation is the best predictor of 1-month survival [15-19].

To identify variables associated with the number of pre-hospital defibrillations or 1-month survival, we performed multiple logistic regression analysis, using the number of defibrillations (0: ≧3, 1: ≦2) or 1-month survival (0: “no”, 1: “yes”) as the dependent variable and the predictors listed in Table 1 (aside from endpoint) as the independent variables. With respect to the time between the first defibrillation and the first ROSC prior to hospitalization, because previous studies reported differences between the initial and recurrent VF during prehospital CPR [1,9], dummy variables were introduced to differentiate these two categories (i.e., 1: ≦1 min, 0: > 1 min). “1: ≦ 1 min” indicates those cases in which a response with a sustained, organized rhythm was obtained using a single shock, whereas “0: > 1 min” indicates all other cases. In order to verify the validity of the initial SDA analysis, we conducted SDA with 1-month survival as the dependent variable and time from the call until arrival at the hospital as a predictor variable in the same sample, and we identified a cut-off point in the time from the call until the arrival at the hospital used to divide the patients into two groups. Then, we calculated the mean shock numbers in the two hospital arrival time groups. Two-tailed p-values < 0.05 were considered significant. Analyses were conducted using SPSS ver. 19 software (SPSS, Inc., Chicago, IL, USA).

### Results

**Figure 2:**
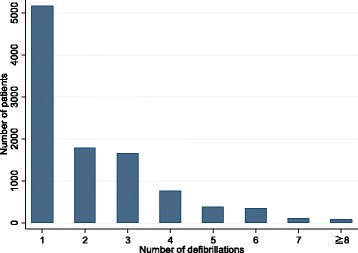
Distribution of patients with OHCA by the number of prehospital defibrillations.

The descriptive characteristics of the study patients with OHCA are summarized in Table 1. The mean age of the study subjects was 65.73 (±15.28) yr. Approximately 79% of the study subjects were male. Overall, 6,480 of the 20,851 OHCA cases survived for at least 1 month (31.08%). The mean number of defibrillation shocks was 2.36 (±1.67). In addition, the number of patients who need 2 or more defibrillation shocks were about 50% of all the study subjects (Figure 2).

**Figure 3:**
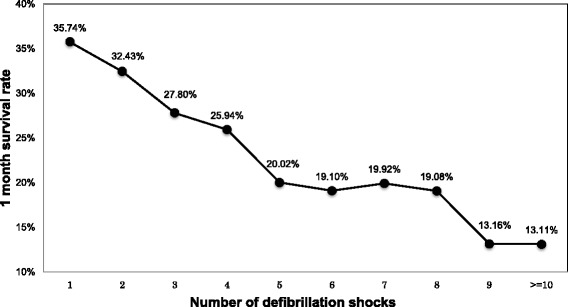
One-month survival of patients with out-of-hospital cardiac arrest (OHCA) according to the number of defibrillation shocks.

Figure 3 shows the 1-month survival rates according to the number of defibrillation shocks. Of the 10 subject groups defined by the number of defibrillation shocks, the 1-month survival rate was highest among subjects who received one shock (35.74%). One-month survival rates in these subject groups decreased as the number of shocks increased, and 1-month survival was lowest among subjects who were delivered 10 or more defibrillation shocks (13.11%).

Table 2 shows the SDA results using 1-month survival rate as a dependent variable and the number of shocks as a predictor variable. Nine cut-off points were used for the number of shocks, and the cut-off point was forwarded by one. Although the χ2 value at every cut-off point was significant (all p < 0.0001), the χ2 value at the cut-off point between two and three shocks was largest (χ2 = 209.61, p < 0.0001). The patients were divided into ≤ 2 shocks (Group 1) and ≥ 3 shocks groups (Group 2) based on the number of defibrillation shocks. Within the ≤ 2 shocks group (Group 1), 34.48% of the patients survived at least 1 month, whereas 24.75% survived at least 1 month within the ≥ 3 shocks group (Group 2).

**Table 2:** **Results of signal detection analysis concerning the association between the number of defibrillation shocks and 1-month survival among patients with out-of-hospital cardiac arrest presenting with ventricular fibrillation**

		**Number and 1-month survival rate (%)**				***χ*** ^***2***^ **value**	***P*** **value**
		**Group 1**		**Group 2**			
1	≤1(Group 1)/≥2(Group 2)	3003	(35.74%)	3477	(27.93%)	142.68	<0.0001
**2**	≤**2(Group 1)/**≥**3(Group 2)**	**4677**	**(34.48%)**	**1803**	**(24.75%)**	**209.61**	**<0.0001**
3	≤3(Group 1)/≥4(Group 2)	5616	(33.15%)	864	(22.11%)	180.63	<0.0001
4	≤4(Group 1)/≥5(Group 2)	6065	(32.48%)	415	(19.06%)	163.82	<0.0001
5	≤5(Group 1)/≥6(Group 2)	6260	(31.86%)	220	(18.29%)	97.50	<0.0001
6	≤6(Group 1)/≥7(Group 2)	6374	(31.48%)	106	(17.49%)	53.78	<0.0001
7	≤7(Group 1) ≥8(Group 2)	6425	(31.34%)	55	(15.71%)	39.23	<0.0001
8	≤8(Group 1)/≥9(Group 2)	6454	(31.25%)	26	(13.13%)	30.06	<0.0001
9	≤9(Group 1)/≥10(Group 2)	6464	(31.18%)	16	(13.11%)	18.49	<0.0001

The patients were divided into ≤ 2 shocks (Group 1) and ≥ 3 shocks groups (Group 2) based on the number of defibrillation shocks, determined by the largest Chi-squared value.

**Figure 4:**
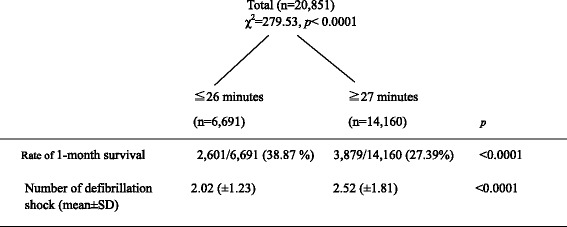
Optimal cutoff time point from the call until arrival at the hospital, which was the best predictor of 1-month survival. The 1-month survival rate and mean number of defibrillation shocks in the two groups divided by the signal detection analysis are also shown.

The time from call until arrival at the hospital was another predictor of 1-month survival in patients with an OHCA. Thus, the cutoff point in the time from the call and arrival at hospital most closely related to 1-month survival was identified by survey documentation and analysis (SDA; Table 3). A total of 47 values were used to represent the time between the call and arrival at the hospital, and the cut-off value was forwarded by one. Although *χ*^*2*^ for 40 values was significant (all *p* < 0.05), the largest value for *χ*^*2*^ was between 26 and 27 minutes (*χ*^*2*^ = 279.53, *p* < 0.0001). Patients were divided into two groups: ≤ 26 minutes (group 1) and ≥ 27 minutes (group 2) based on the time elapsed from the call until arrival at the hospital. The 1-month survival rate and the mean number of shocks were 38.87% and 2.02 (±1.23), respectively, in the ≤ 26 min group and 27.3% and 2.52 (±1.81), respectively, in the ≥ 27 min group (Figure 4). The mean number of shocks in the ≤ 26 min group was very close to two (i.e., 2.02).

**Table 3:** **Results of signal detection analysis concerning the association between the time (min.) from the call to arrival at the hospital and 1-month survival among patients with out-of-hospital cardiac arrest presenting with ventricular fibrillation**

		**Number and 1-month survival rate (%)**				***χ*** ^***2***^ **value**	***P*** **value**
		**Group 1**		**Group 2**			
1	≤4(Group 1)/≥5(Group 2)	0	(0.00%)	6480	(31.08%)	0.45	0.5019
2	≤5(Group 1)/≥6(Group 2)	0	(0.00%)	6480	(31.08%)	0.90	0.3423
3	≤6(Group 1)/≥7(Group 2)	1	(25.00%)	6479	(31.08%)	0.07	0.7928
4	≤7(Group 1)/≥8(Group 2)	6	(60.00%)	6474	(31.06%)	3.91	0.0481
5	≤8(Group 1)/≥9(Group 2)	10	(66.67%)	6470	(31.05%)	8.88	0.0029
6	≤9(Group 1)/≥10(Group 2)	18	(54.55%)	6462	(31.04%)	8.50	0.0036
7	≤10(Group 1) ≥11(Group 2)	26	(56.52%)	6454	(31.02%)	13.93	0.0002
8	≤11(Group 1)/≥12(Group 2)	38	(52.05%)	6442	(31.00%)	15.05	<0.0001
9	≤12(Group 1)/≥13(Group 2)	58	(47.54%)	6422	(30.98%)	15.53	<0.0001
10	≤13(Group 1)/≥14(Group 2)	85	(50.00%)	6395	(30.92%)	28.65	<0.0001
11	≤14(Group 1)/≥15(Group 2)	142	(50.71%)	6338	(30.81%)	51.09	<0.0001
12	≤15(Group 1) ≥16(Group 2)	220	(50.00%)	6260	(30.67%)	75.14	<0.0001
13	≤16(Group 1)/≥17(Group 2)	336	(50.15%)	6144	(30.44%)	117.55	<0.0001
14	≤17(Group 1)/≥18(Group 2)	453	(46.85%)	6027	(30.31%)	117.71	<0.0001
15	≤18(Group 1)/≥19(Group 2)	614	(45.92%)	5866	(30.06%)	147.00	<0.0001
16	≤19(Group 1)/≥20(Group 2)	800	(44.57%)	5680	(29.81%)	166.88	<0.0001
17	≤20(Group 1) ≥21(Group 2)	966	(43.83%)	5514	(29.57%)	187.09	<0.0001
18	≤21(Group 1)/≥22(Group 2)	1234	(43.54%)	5246	(29.12%)	237.92	<0.0001
19	≤22(Group 1)/≥23(Group 2)	1469	(42.25%)	5011	(28.84%)	243.13	<0.0001
20	≤23(Group 1)/≥24(Group 2)	1693	(40.23%)	4787	(28.76%)	206.30	<0.0001
21	≤24(Group 1)/≥25(Group 2)	1985	(39.80%)	4495	(28.34%)	232.64	<0.0001
22	≤25(Group 1) ≥26(Group 2)	2269	(39.15%)	4211	(27.97%)	244.07	<0.0001
**23**	**≤26(Group 1)/≥27(Group 2)**	**2601**	**(38.87%)**	**3879**	**(27.39%)**	**279.53**	**<0.0001**
24	≤27(Group 1)/≥28(Group 2)	2863	(37.78%)	3617	(27.25%)	249.38	<0.0001
25	≤28(Group 1)/≥29(Group 2)	3164	(37.11%)	3316	(26.91%)	244.74	<0.0001
26	≤29(Group 1)/≥30(Group 2)	3446	(36.69%)	3034	(26.47%)	251.69	<0.0001
27	≤30(Group 1) ≥31(Group 2)	3683	(36.14%)	2797	(26.24%)	238.47	<0.0001
28	≤31(Group 1)/≥32(Group 2)	3929	(35.52%)	2551	(26.06%)	216.89	<0.0001
29	≤32(Group 1)/≥33(Group 2)	4178	(35.16%)	2302	(25.67%)	215.18	<0.0001
30	≤33(Group 1)/≥34(Group 2)	4380	(34.63%)	2100	(25.60%)	189.41	<0.0001
31	≤34(Group 1)/≥35(Group 2)	4545	(34.33%)	1935	(25.42%)	179.13	<0.0001
32	≤35(Group 1) ≥36(Group 2)	4714	(33.91%)	1766	(25.41%)	156.33	<0.0001
33	≤36(Group 1)/≥37(Group 2)	4865	(33.56%)	1615	(25.41%)	137.16	<0.0001
34	≤37(Group 1)/≥38(Group 2)	4998	(33.23%)	1482	(25.51%)	116.66	<0.0001
35	≤38(Group 1)/≥39(Group 2)	5145	(32.94%)	1335	(25.53%)	100.46	<0.0001
36	≤39(Group 1)/≥40(Group 2)	5262	(32.64%)	1218	(25.75%)	81.05	<0.0001
37	≤40(Group 1) ≥41(Group 2)	5356	(32.37%)	1124	(26.12%)	62.18	<0.0001
38	≤41(Group 1)/≥42(Group 2)	5437	(32.16%)	1043	(26.43%)	49.20	<0.0001
39	≤42(Group 1)/≥43(Group 2)	5507	(31.91%)	973	(27.07%)	32.51	<0.0001
40	≤43(Group 1)/≥44(Group 2)	5583	(31.74%)	897	(27.52%)	22.78	<0.0001
41	≤44(Group 1)/≥45(Group 2)	5637	(31.60%)	843	(27.96%)	15.99	<0.0001
42	≤45(Group 1) ≥46(Group 2)	5703	(31.44%)	777	(28.67%)	8.42	0.0037
43	≤46(Group 1)/≥47(Group 2)	5766	(31.38%)	714	(28.83%)	6.66	0.0099
44	≤47(Group 1)/≥48(Group 2)	5810	(31.25%)	670	(29.63%)	2.47	0.1159
45	≤48(Group 1)/≥49(Group 2)	5856	(31.22%)	624	(29.84%)	1.66	0.1981
46	≤49(Group 1)/≥50(Group 2)	5900	(31.15%)	580	(30.32%)	0.57	0.4518
47	≤50(Group 1)/≥51(Group 2)	5949	(31.15%)	531	(30.33%)	0.50	0.4774

Patients were divided into two groups: ≤ 26 minutes (group 1) and ≥ 27 minutes (group 2) based on the time elapsed from the call until arrival at the hospital, determined by the largest Chi-squared value.

Table 4 shows the characteristics of the patients with OHCA categorized by the number of defibrillation shocks (i.e., ≤ 2 and ≥ 3 shocks group). Significant differences were observed between the two groups in all study variables except for “rescue breathing”. The time from the call until arrival at the hospital was also significantly longer in the ≥3 shocks group than the ≤2 shocks group (35.02. *vs*. 32.96 min., p < 0.0001). The rates of use of an ALS device and epinephrine were also significantly higher in the ≥3 shocks group than the ≤2 shocks group (p < 0.0001). Thus, the time from the call until arrival at the hospital in the ≥3 shocks group, which had more events (*i.e.,* more shocks, ALS and epinephrine), was longer than that in the ≤2 shocks group, which had fewer events. Because a significant difference in the study variables was detected between the two defibrillation shock groups (Table 4), multiple logistic regression analysis was performed to identify the factors associated with the number of defibrillation shocks (0: ≥ 3, 1: ≤ 2; Table 5). Of the independent variables, the origin of OHCA (OR = 0.54, 95% CI: 0.41, 0.71), advanced MD life support (OR = 0.69, 95% CI: 0.58, 0.83), use of ALS devices (OR = 0.63, 95% CI: 0.55, 0.73) use of epinephrine (OR = 0.37, 95% CI: 0.32, 0.44) and time between the first defibrillation and the first ROSC prior to hospitalization (OR = 3.08, 95% CI: 2.38, 3.99) were associated significantly with the number of defibrillations. With respect to the time between the first defibrillation and the first ROSC prior to arrival at the hospital, compared with patients in which ROSC was not obtained in response to the first defibrillation (i.e., ≧ 2 min), those in which a ROSC response was obtained (i.e., ≦ 1 min) were 3.06-fold more likely to have received ≦ 2 defibrillations.

**Table 4:** **Characteristics of out-of-hospital cardiac arrest (OHCA) patients who received biphasic defibrillation treatment before hospital arrival according to the number of defibrillations**

**Variable**	**≤2 times (** ***n =*** **13,565)**	**≥3 times (** ***n =*** **7,286)**	***P*** **-value**
(OHCA patients)			
Age (yr) (mean ± standard deviation [SD])	66.19 ± 15.32	64.09 ± 15.07	<.0001
Sex (male), no. (%)	10430 (76.89)	5938 (81.50)	<.0001
Origin of OHCA (cardiac origin), no. (%)	11863 (87.45)	6735 (92.44)	<.0001
Relationship between bystander and patient (family member), no. (%)	6997 (51.58)	4197 (57.60)	<.0001
(CPR initiated by bystander)			
Chest compressions (yes), no. (%)	6167 (45.46)	3514 (48.23)	<.0001
Rescue breathing (yes), no. (%)	1446 (10.66)	782 (10.73)	.8705
(Life support by EMS personnel)			
Emergency life-saving technician in ambulance (yes), no. (%)	13169 (97.08)	7115 (97.65)	.0154
Advanced life support by MD (yes), no. (%)	1628 (12.00)	1020 (14.00)	<.0001
Time from call to arrival at scene (min) (mean ± SD)	7.05 ± 3.24	7.17 ± 3.27	.0159
Time from call to arrival at hospital (min) (mean ± SD)	32.96 ± 15.88	35.02 ± 14.67	<.0001
Use of ALS devices (laryngeal mask/an adjunct airway/ tracheal tubes), no. (%)	4946 (36.46)	3494 (47.95)	<.0001
Epinephrine use (yes), no. (%)	2207 (16.27)	2437 (33.45)	<.0001
Time from the first defibrillation to the first ROSC before hospital arrival (min) (mean ± SD)	6.27 ± 9.63	11.91 ± 8.09	<.0001
Number of defibrillations by EMS personnel (mean ± SD)	1.38 (0.49)	4.18 (1.55)	<.0001
Proportions of the number of defibrillations (%)			<.0001
1	8403 (61.95)	0 (0.00)	
≥2	5162 (38.05)	7286 (100.00)	
Frequency of patients who took ≤1 min. from the first defibrillation to the first ROSC before hospital arrival (%)	2393 (17.64)	350 (4.81)	<.0001
(Endpoint)			
1-month survival after cardiac arrest (yes), no. (%)	4677 (34.48)	1803 (24.75)	<.0001
ROSC (yes), no. (%)	5188 (38.25)	1688 (23.17)	<.0001

**Table 5:** **Results of logistic regression analysis: adjusted odds ratios (ORs) and 95% confidence intervals (CIs) for the factors related to the number of defibrillation shocks**

**Variables**	**Odds ratio**	**95% CI**
Age	1.01	(1.01–1.02)
Sex (0: female, 1: male)	0.84	(0.72–0.98)
Origin of OHCA (0: non cardiac, 1: cardiac)	0.54	(0.41–0.71)
Relationship between bystander and patient (family member), no. (%)	0.96	(0.85–1.09)
Chest compressions (0: no, 1: yes)	0.93	(0.81–1.06)
Rescue breathing (0: no, 1: yes)	0.97	(0.79–1.18)
Emergency life-saving technician in ambulance (0: no, 1: yes)	1.30	(0.83–2.03)
Advanced life support by MD (0: no, 1: yes)	0.69	(0.58–0.83)
Time from call to arrival at scene (min)	1.01	(0.99–1.03)
Time from call to arrival at hospital (min)	0.99	(0.99–1.00)
Use of ALS devices (0: no, 1: laryngeal mask/an adjunct airway/ tracheal tubes)	0.63	(0.55–0.73)
Epinephrine use (yes), no. (%)	0.37	(0.32-0.44)
Time from the first defibrillation to the first ROSC before hospital arrival (0: ≥2 min, 1: ≤1 min)	3.08	(2.38–3.99)
*Hosmer–Lemeshow* test, *χ2* = 3.21 (p = 0.92)		

Note: The effects of the 47 prefectures in Japan were controlled for by introducing 46 dummy variables in the analysis.

Finally, multiple logistic regression analysis was used to calculate the adjusted ORs of the study variables, including those for the shock groups, to evaluate their effects on 1-month survival (Table 6). The number of defibrillations was a significant predictor of 1-month survival when the effects of the other relevant variables were controlled for (OR = 1.19, 95% CI: 1.03, 1.38). Notably, other variables, such as origin of OHCA (OR = 2.81, 95% CI: 2.26, 3.49), use of ALS devices (OR = 0.68, 95% CI: 0.59, 0.79), use of epinephrine (OR = 0.33, 95% CI: 0.28, 0.39), and time between the first defibrillation and the first ROSC (OR = 1.45, 95% CI: 1.18, 1.78), were more closely related to 1-month survival compared with the number of defibrillations. Compared with patients who did not use ALS devices, those who did were 0.68-fold less likely to survive at least 1 month after the event. OHCA patients who received epinephrine were 0.33-fold less likely to survive at least 1 month after the event than were those who did not.

**Table 6:** **Results of logistic regression analysis: adjusted odds ratios (ORs) and 95% confidence intervals (CIs) for the factors related to one-month survival**

**Variables**	**Odds ratio**	**95% CI**
Age	0.97	(0.96–0.97)
Sex (0: female, 1: male)	1.18	(1.02–1.37)
Origin of OHCA (0: non cardiac, 1: cardiac)	2.81	(2.26–3.49)
Relationship between bystander and patient (family member), no. (%)	0.77	(0.68–0.88)
Chest compressions (0: no, 1: yes)	1.21	(1.06–1.38)
Rescue breathing (0: no, 1: yes)	1.07	(0.86–1.32)
Emergency life-saving technician in ambulance (0: no, 1: yes)	1.32	(0.87–2.02)
Advanced life support by MD (0: no, 1: yes)	0.80	(0.67–0.95)
Time from call to arrival at scene (min)	0.96	(0.94–0.98)
Time from call to arrival at hospital (min)	0.99	(0.93–0.99)
Use of ALS devices (0: no, 1: laryngeal mask/an adjunct airway/ tracheal tubes)	0.68	(0.59–0.79)
Epinephrine use (yes), no. (%)	0.33	(0.28-0.39)
Time from the first defibrillation to the first ROSC before hospital arrival (0: ≥2 min, 1: ≤1 min)	1.45	(1.18–1.78)
Number of defibrillations (0: ≥3, 1: ≤2)	1.19	(1.03–1.38)
*Hosmer–Lemeshow* test, *χ2* = 11.71 (p = 0.17)		

Note: The effects of the 47 prefectures in Japan were controlled for by introducing 46 dummy variables in the analysis.

There were 6480 survivors 1 month after an event. Regarding the neurologic status of the survivors, the mean CPC score was 1.93 (±1.27), and the proportion of survival with minimal neurological impairment [CPC (1,2)] was 69.46%. The mean OPC score was 1.95 (±1.27), and the proportion of survival with minimal neurological impairment [OPC (1,2)] was 68.83%. With respect to the proportions of CPC (1, 2) and OPC (1, 2), there was a significant difference between the ≤2 shocks and the ≥3 shocks groups (CPC, 70.69% in the ≤2 shocks group, and 66.2% in the ≥3 shocks groups; OPC, 69.96% in the ≤2 shocks group, and 65.9% in the ≥3 shocks groups) (p < 0.01, respectively). With respect to the mean CPC and OPC scores, there was a significant difference between the ≤2 shocks and the ≥3 shocks groups (CPC, 1.89 (±1.26) in the ≤2 shocks group, and 2.02 (±1.30) in the ≥3 shocks groups; OPC, 1.92 (±1.26) in the ≤2 shocks group, and 2.04 (±1.29) in the ≥ 3shocks groups), with p < 0.01, respectively.

## Discussion

In patients with an OHCA who were resuscitated following the 2005 or 2010 resuscitation guideline, the association between the number of pre-hospitalization defibrillations and long-term survival has not been evaluated. We examined the association between the number of defibrillations and 1-month survival. Although the 2005 and 2010 Japanese resuscitation guidelines are similar to the 2010 AHA Guidelines for CPR and ECC, emergency lifesaving technicians must be granted approval by an on-line emergency physician before delivering second and third shocks during pre-hospitalization emergency care; this is because the Japanese Medical Practitioner’s Law prohibits medical treatment by anyone other than medical doctors [20]. Therefore, it should be noted that the following comments primarily relates to prehospital CPR in Japan, and our findings are generalizable only when identical regulation systems are in place. In addition, no cause-effect associations could be assumed between the number of shocks, the use of airway devices used and epinephrine administered, and mortality, because of the observational nature of the study.

First of all, the cutoff point in the number of pre-hospitalization defibrillations most closely associated with 1-month survival was between two and three, using the 2005 and 2010 guidelines (Table 2). Specifically, patients with OHCA receiving one or two shocks were 1.19-fold more likely to survive for at least 1 month compared with those who were delivered three or more shocks in the field (Table 6). The endpoint of the 1-month survival might be affected by many confounding factors, such as in-hospital treatment. However, this information was not available in the study. Thus, a sensitivity analysis of the association between the number of shocks and intermediate endpoints, such as survival of CA before in-hospital admission (i.e., ROSC before arrival at the hospital) was evaluated (Additional file 1: Table S1). The cutoff point for the number of pre-hospitalization defibrillations most closely associated with ROSC before hospital arrival was also between 2 and 3, which suggests that 2 or less defibrillations is related to the better resuscitation outcome of patients with an OHCA. A Norwegian study on the association between the quality of CPR for patients with OHCA and outcomes reported that the median number of shocks among the study subjects was two under the 2005 resuscitation guidelines [21]. Our findings are the first on the association between the number of defibrillation shocks and 1-month survival under the 2005 and 2010 guidelines.

We refer to the practical implications of the finding. Since implementing the 2005 and 2010 guidelines, which resulted in decreased post-shock time and increased CPR time, increases in the rate of recurrence and the duration of VF have been reported [22]. More than one defibrillation often is necessary during prehospital care because recurrence of shockable rhythm after the first defibrillation is common [1,2], and more than half of OHCA under the 2005 guidelines are suggested to be in VF when heart rhythm is checked 2 min after the first shock [7]. As the Medical Practitioners Law prohibits medical treatment by anyone other than medical doctors in Japan [20], emergency lifesaving technicians must have the approval of an on-line emergency physician to deliver the second or third shocks in pre-hospital emergency care. Thus, when two or more shocks are needed, this will take longer in Japan than in areas where permission from an on-line emergency physician is not required under the 2005 and 2010 resuscitation guidelines. In resuscitation of patients with an OHCA, it might be necessary to consider allowing delivery of two or more shocks without approval from an on-line emergency physician before arriving at the hospital.

The origin of OHCA, use of ALS devices, use of epinephrine, and time between the first defibrillation and the first ROSC were more closely associated with 1-month survival than were the number of defibrillations (Table 6). Concerning ALS devices, in a propensity score–matched Japanese cohort study, the adjusted ORs of neurologically favorable survival outcomes were significantly lower both for endotracheal intubation (adjusted OR = 0.45, 95% CI: 0.37, 0.55) and use of supraglottic airways (adjusted OR = 0.36, 95% CI: 0.33, 0.39) [23]. These findings are consistent with those of the present study. Negative influences of ALS might be due to the following reasons [24]. First, the time taken to perform a tracheal intubation might lead to ineffective chest compressions with significant interruptions. Second, tracheal intubation requires a high level of technical skill, and the success rate is low. Failure is associated with a number of major complications. Third, after tracheal intubation, unintentional hyperventilation increases intrathoracic pressure, resulting in decreased coronary and cerebral perfusion pressure. In another Japanese study, epinephrine use was negatively associated with 1-month survival (OR = 0.60, 95% CI: 0.49, 0.74) [25]. This result is in accordance with those of the present study. As for relationship between bystander and patient, OHCAs handled by multiple rescuers were associated with higher incidences of bystander CPR but were not associated with better outcomes among OHCAs that occurred at home [26]. Present finding is similar to this finding. More importantly, variables other than the number of defibrillations (i.e., origin of OHCA, use of ALS devices, epinephrine use, time between the first defibrillation and first ROSC, and others) were associated more closely with 1-month survival, implying that the number of defibrillations might be relatively unimportant with respect to the long-term outcome of OHCA patients.

Several notable points should be emphasized from our study. First, compared with previous studies reporting that survival after VF arrest is <20% [27,28], the 1-month survival rate after cardiac arrest was very high (i.e., 31.08%) (Table 1). This was due to the multiple selection criteria of the study subjects, such as age (18–110 years), time from call until scene arrival (≤60 min), time from call until arrival at a hospital (≤480 min), eyewitness (yes), initial rhythm (VF/pulseless VT), use of public AED (no), and defibrillation by ELS personnel using a biphasic defibrillator (yes). Second, 1-month survival rate decreased as the number of shocks increased (Figure 3). A previous study revealed that a large number of VF recurrences leads to lower patient survival due to cardiac arrest [1]. Thus, the present finding was consistent with the previous findings. Notably, in the figure, a non-significant peak in the 1-month survival rate was observed around where the number of shocks was 7 (Figure 3). Previous studies have reported similar findings, and this might be due to episodes with several min of good quality CPR [29-31]. Third, although there was a significant difference in the mean CPC and OPC scores between the ≤2 shocks and the ≥3 shocks groups, the mean CPC and OPC scores were more or less in category 2, indicating patients with moderate cerebral disability. This might be due to the analysis methods with subjects limited to survivors at 1 month after the event. Fourth, since Japanese EMS have a limitation to administer the second and following shocks to cardiovert a patient with a shockable rhythm, we compared outcomes between patient groups that needed ≥1 and 1 shocks. There was a significant difference between the 1 and ≥2 shock groups with respect to the proportions of ROSC (positive; 40.51% *vs.* 27.89%), 1-month survival (positive; 35.73% *vs*. 27.93%), CPC (1, 2) (26.00% *vs.* 18.94%), and OPC (1, 2) (26.78% *vs.* 18.75%) with p < 0.01, respectively. The on-line control by physicians could be one explanation for the significant differences in outcome measures between the 1 and ≥2 shock groups in Japan.

We refer to a methodological point in the study. As the SDA is an exploratory technique, the present findings need to be verified. Time from call until arrival at the hospital was another predictor of 1-month survival in patients with OHCA. Thus, SDA was applied to the data, and an optimal cut-off time point from call to arrival at the hospital was identified to predict 1-month survival. Among the higher survival rate group predicted by the time from call until arrival at the hospital (≤26 min; 1-month survival rate 38.87%), the mean number of shocks was 2.02 (Figure 4). In summary, the numbers of shocks in the high survival group in the first and second SDA were similar. The results are based on two different approaches and consistently show that a distinct cutoff point exists between two and three shocks. Thus, the SDA result (i.e., the cut-off point between two and three shocks is the best predictor of 1-month survival among patients with OHCA) is supported, and we believe that the present findings can be trusted.

Our study had several limitations. First, data regarding the use of cardio active and antirrhythmic agents were not included in the analysis because of the lack of the data. Second, data on in-hospital CPR after arrival at the hospital were not included in the analysis. Some of our findings were due to factors associated with in-hospital resuscitation, such as hypothermia [32] and mechanical chest compression devices [33]. We could not find a specific reason that there should be a difference between the ≤ 2 shocks and ≥ 3 shocks groups with respect to in-hospital care. However, it is possible that the quality of in-hospital resuscitation may have influenced 1-month survival after the event. We admit that the effects of such factors were not controlled in this study. Third, although the 2005 and 2010 resuscitation guidelines were quite similar to the 2010 AHA Guidelines for CPR and ECC, permission from an on-line emergency physician is required when the second or further defibrillation shocks are necessary in Japan. Thus, the external validity of the present findings might be limited. Fourth, we identified the cutoff point in the number of shocks to predict 1-month survival after OHCA in adults, but caution should be taken when attributing a causal relationship between the predictor and the proportion of patients surviving. Fifth, there was a high exclusion rate due to the numerous inclusion criteria. Specifically, of the initial number of cardiac arrest cases between January 1, 2009 and December 31, 2012 (n = 493,320), only 4.2% of cases were used in this analysis. Sixth, due to a lack of information, it was not possible to specify the amount of time that second defibrillations were delayed to allow for MD approval. In a future study, an evaluation of significant differences in survival based on the time to the second defibrillation should be conducted.

## Conclusions

In summary, the cutoff point in the number of defibrillations of patients with an OHCA most closely related to 1-month survival was between 2 and 3, and when ≥3 shocks were needed, the likelihood of non-survival 1 month after an OHCA increased. Because of the observational nature of this study, further research is necessary to verify these findings.

## Additional file

Additional file 1: Table S1. Results of signal detection analysis concerning the association between the number of defibrillation shocks and ROSC among patients with out-of-hospital cardiac arrest presenting with ventricular fibrillation.
