# Supplementary material for: The number of prehospital defibrillation shocks and 1-month survival in patients with out-of-hospital cardiac arrest
Source: Scand J Trauma Resusc Emerg Med. 2015 Apr 17;23:34. doi: 10.1186/s13049-015-0112-4 (PMC4404114; doi:10.1186/s13049-015-0112-4)
Supplement: Additional file 1: Table S1. — Results of signal detection analysis concerning the association between the number of defibrillation shocks and ROSC among patients with out-of-hospital cardiac arrest presenting with ventricular fibrillation. [file 13049_2015_112_MOESM1_ESM.docx]

## Additional file 1: Table S1. Results of signal detection analysis concerning the association between the number of defibrillation shocks and ROSC among patients with out-of-hospital cardiac arrest presenting with ventricular fibrillation.

|  |  | Number and 1-month survival rate (%) | | | | *χ^2^* value | *P* value |
| --- | --- | --- | --- | --- | --- | --- | --- |
|  |  | Group 1 | | Group 2 | |  |  |
| 1 | ≤1(Group 1)/≥2(Group 2) | 3404 | (40.51%) | 3472 | (27.89%) | 361.33 | <0.0001 |
| **2** | ≤**2(Group 1)/**≥**3(Group 2)** | 5188 | **(38.25%)** | 1688 | **(23.17%)** | **487.55** | **<0.0001** |
| 3 | ≤3(Group 1)/≥4(Group 2) | 6109 | (36.06%) | 767 | (19.63%) | 387.84 | <0.0001 |
| 4 | ≤4(Group 1)/≥5(Group 2) | 6487 | (34.74%) | 389 | (17.87%) | 251.04 | <0.0001 |
| 5 | ≤5(Group 1)/≥6(Group 2) | 6670 | (33.95%) | 206 | (17.12%) | 145.17 | <0.0001 |
| 6 | ≤6(Group 1)/≥7(Group 2) | 6769 | (33.44%) | 107 | (17.66%) | 66.28 | <0.0001 |
| 7 | ≤7(Group 1) ≥8(Group 2) | 6813 | (33.23%) | 63 | (18.00%) | 36.13 | <0.0001 |
| 8 | ≤8(Group 1)/≥9(Group 2) | 6843 | (33.13%) | 33 | (16.67%) | 24.06 | <0.0001 |
| 9 | ≤9(Group 1)/≥10(Group 2) | 6857 | (33.08%) | 19 | (15.57%) | 16.82 | <0.0001 |
